# Smart Biomaterials for Delivery of Drugs and Cells

**DOI:** 10.34133/bmr.0227

**Published:** 2025-07-31

**Authors:** Seong-Jong Kim, Byeongmoon Jeong, Ki Dong Park, Sei Kwang Hahn, Insup Noh

**Affiliations:** ^1^ Department of Materials Science and Engineering, Pohang University of Science and Technology (POSTECH), 77 Cheongam-ro, Nam-gu, Pohang, Gyeongbuk 37673, South Korea.; ^2^ Department of Chemistry and Nanoscience, Ewha Womans University, 52 Ewhayeodae-gil, Seodaemun-gu, Seoul 03760, South Korea.; ^3^ Department of MolecularScience and Technology, Ajou University, Suwon 16499, South Korea.; ^4^ Convergence Institute of Biomedical Engineering and Biomaterials, Seoul National University of Science and Technology, Seoul 01811, South Korea.

The World Biomaterials Congress (WBC; WBC2024) in Daegu, Korea is the 12th Congress event that has been taking place every 4 years since the first WBC in Vienna, Austria. The event gathers in-person worldwide biomaterial scientists from diverse academies and industries, presenting and sharing new ideas and networking and visiting the industrial exhibitions every 4 years by rotating its host society from the societies in America, Asia, and Europe. However, the recent COVID-19 altered our lives and the way of working worldwide, activating online meetings for WBC2020. These online meetings have made difficult in-person scientific and social activities that are important to biomaterial researchers and industries. It was a great time for the biomaterials society to resume its scientific and social events in WBC2024, Daegu, Korea. The WBC2024 was very successful for the participating biomaterial scientists and engineers in both scientific and social aspects. To foster scientific activities and acknowledge the successful WBC2024, we designed and acknowledged the WBC2024 special issues. The WBC2024 committee invited 4 journals for special issues including Biomaterials Research, Biomaterials Science, Journal of Biomedical Materials Part A, and Regenerative Biomaterials with their specific topics. The journals invite the authors who present their research in the WBC2024, and manuscripts can be submitted until the deadline designated by each journal. A guide for authors and other relevant information for submission of manuscripts are available on each journal’s website.

In this Special Issue of Biomaterials Research, we overview the current state-of-the-art research on the emerging smart biomaterials for delivery of drugs and cells for further clinical applications (Figure) [1]. More specifically, this issue highlights recent advances in bioimaging, theranosis, drug delivery, and tissue microenvironment engineering, showcasing how multifunctional biomaterials are driving next-generation diagnostic, therapeutic, and regenerative technologies.

**Figure.**
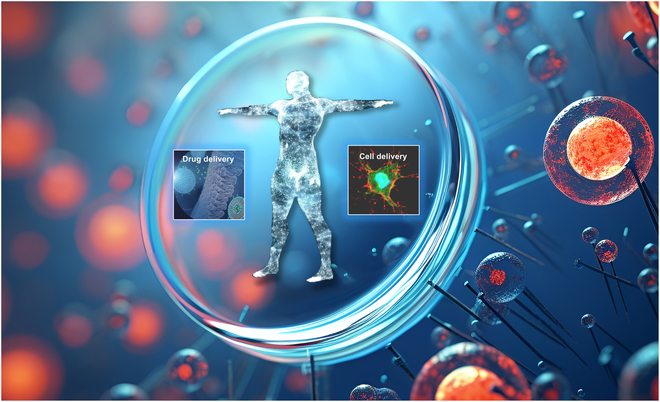
Schematic illustration of smart emerging biomaterials for the delivery of drugs and cells for biomedical applications to bioimaging, theranosis, drug delivery, and tissue microenvironment engineering. Reproduced with permission [1]: Copyright 2015 John Wiley & Sons.

We begin the Special Issue with the history of International Union of Societies for Biomaterials Science and Engineering (IUSBSE), which is a part of the history of the field of biomaterials science. The current president of IUSBSE, J. Kohn, describes the beginnings of IUSBSE from 1980 to 1996 and the growth and activities of IUSBSE from 1996 to 2024.

For the topic of bioimaging and the theranostic applications, Moon et al. [2] use high-frequency optical coherence elastography to noninvasively quantify gingival tissue stiffness and its dynamic response to physiological stimuli. Their work provides a critical diagnostic tool for monitoring soft tissue health and validating the biomechanical compatibility of emerging biomaterials. Choi et al. [3] develop water-dispersible upconversion nanoparticles based on hyaluronic acid–polycaprolactone hybrids that enable deep-tissue imaging and transdermal light-triggered activation using low-power red light. This platform addresses key limitations of visible-light photomedicine and enhances noninvasive delivery strategies. Park et al. [4] present a bifunctional tumor-targeting bioprobe that combines real-time near-infrared (NIR) fluorescence imaging with synergistic photodynamic and photothermal therapy, offering tumor-specific accumulation, reactive oxygen species and heat generation, and in vivo complete decomposition postirradiation. In parallel, Yun et al. [5] demonstrate that precisely controlled mild hyperthermia induced by silica-coated gold nanorods (AuNR@SiO_2_) enhances immune checkpoint blockade therapy with NIR fluorescence imaging, achieving complete tumor regression and long-term immunological memory in vivo.

For drug delivery systems, Yang et al. [6] present an NIR-triggered supramolecular hydrogels containing MXene–doxorubicin complexes. Their system achieves the synergistic chemo–photothermal therapy with excellent tumor localization and spatiotemporal control over drug release. Lee et al. [7] investigate the critical role of poly(ethylene glycol) (PEG) lipid structure and ratio in repeated administrations of mRNA-loaded lipid nanoparticles. Their results highlight that while ionizable lipids have minimal impact, PEG strongly influences protein expression efficiency and immunogenicity, offering an important strategy to avoid the accelerated blood clearance during chronic dosing. Complementing these experimental advances, Mao and Yoo [8] review the design and functionalization of inorganic nanoparticles (INPs) for cancer immunotherapy, highlighting their versatility for targeted delivery, immune activation, and combination therapy. With tunable surfaces and multifunctional payload capacity, INPs are emerging as the next-generation platforms for precision drug delivery and adaptive, patient-tailored treatment strategies.

Last, in tissue engineering and microenvironment modeling, Han et al. [9] develop a cerebrovascular-specific extracellular matrix bioink by blending brain- and vessel-derived decellularized matrices. This bioink enables the fabrication of tubular, perfusable blood–brain barrier constructs via coaxial 3-dimensional (3D) bioprinting. The model demonstrates spontaneous self-assembly of endothelial and pericyte layers, robust junction formation, and neuroinflammatory responsiveness, thereby offering a physiologically relevant modeling of neuroinflammation-related pathologies. Patel et al. [10] develop a cytocompatible cryopreservation strategy using low-molecular-weight PEGs for 3D stem cell spheroids, overcoming the toxicity issue of conventional cryoprotectants while preserving cell viability and function. Yi et al. [11] demonstrate that electrical stimulation at optimized low-voltage conditions enhances the chondrogenic differentiation of human mesenchymal stem cells (MSCs). The electrically primed MSCs significantly increased cartilage-specific gene expression, proteoglycan deposition, and in vivo regeneration in a rat osteochondral defect model, offering a streamlined alternative to growth-factor-based preconditioning. Cho et al. [12] demonstrate that MSCs with elevated glutathione levels achieved by human platelet lysate priming secrete insulin-like growth factor 2 (IGF2), which alleviates osteoarthritis by rejuvenating senescent chondrocytes. IGF2 promotes autophagy and matrix production, and its knockdown abolishes these therapeutic effects, highlighting its key paracrine role.

In summary, this WBC2024 Special Issue under the title of smart biomaterials for delivery of drugs and cells provides the current state-of-the-art research on emerging multifunctional nanomaterials for diverse biomedical applications to bioimaging, theranosis, drug delivery, and tissue microenvironment engineering, providing perspectives on the technological challenges and future research directions. We hope that this special issue can make a great impact on the biomaterials community to diversify from the conventional materials and systems to the innovative healthcare materials and systems for futuristic biomedical applications.
